# Periquiteira (*Cochlospermum orinocense*): A Promising Amazon Fiber for Application in Composite Materials

**DOI:** 10.3390/polym15092120

**Published:** 2023-04-28

**Authors:** Miriane Alexandrino Pinheiro, Maurício Maia Ribeiro, Diemison Lira Santa Rosa, Damares da Cruz Barbosa Nascimento, Alisson Clay Rios da Silva, Marcos Allan Leite dos Reis, Sergio Neves Monteiro, Verônica Scarpini Candido

**Affiliations:** 1 Engineering of Natural Resources of the Amazon Program, Federal University of Pará—UFPA, Belem 66075-110, Brazil; 2 Materials Science and Engineering Program, Federal University of Pará—UFPA, Ananindeua 67130-660, Brazil; 3 Materials Science Program, Military Institute of Engineering—IME, Rio de Janeiro 22290-270, Brazil

**Keywords:** natural lignocellulosic fiber, periquiteira fiber, characterization

## Abstract

Natural lignocellulosic fibers (NLFs) have in recent decades appeared as sustainable reinforcement alternatives to replace synthetic fibers in polymer composite material applications. In this work, for the first time, the periquiteira (*Cochlospermum orinocense*), a lesser known NLF from the Amazon region, was analyzed for its density and, by X-ray diffraction (XRD), to calculate the crystallinity index as well as the microfibrillar angle (MFA), thermogravimetric analysis (TGA), Fourier transform infrared spectroscopy (FTIR), scanning electron analysis (SEM) and tensile strength. The apparent density found for the periquiteira fiber was 0.43 g/cm^3^, one of the NLF’s lowest. XRD analysis indicated a crystallinity index of 70.49% and MFA of 7.32°. The TGA disclosed thermal stability up to 250 °C. The FTIR analysis indicated the presence of functional groups characteristic of NLFs. The SEM morphological analysis revealed that the periquiteira fiber presents fine bundles of fibrils and a rough surface throughout its entire length. The average strength value of the periquiteira fiber was found as 178 MPa. These preliminary results indicate that the periquiteira fiber has the potential to be used as a reinforcing agent in polymeric matrices and can generate a lightweight composite with excellent mechanical properties that can be used in various industrial sectors.

## 1. Introduction

Sustainable development and ecological responsibility have, in recent years, induced the search for alternative, cost-effective, natural materials that can be used as a reinforcement of high-performance composites and aggregate value to these materials [1,2,3,4]. Conventional composites, usually applied in several technological sectors, are produced with synthetic reinforcement materials. However, the production of these materials requires greater manufacturing energy, in addition to generating a large amount of non-degradable waste after its lifetime, causing pollution to the environment [5,6].

The search for natural, ecologically correct, sustainable materials that offer mechanical properties comparable to synthetic materials, and which can be produced at a lower cost, has increased considerably. In this context, natural lignocellulosic fibers (NLFs) and fabrics emerge as an alternative use to replace their synthetic equivalents, such as glass, carbon and Kevlar fibers [3,4,5,6]. This replacement is occurring in several industrial sectors, such as the automotive, ballistic armor, sports and of construction industries [7,8,9,10].

When compared to synthetic fibers, NLFs are generally lighter, cheaper and have specific properties for similar applications [11,12,13,14,15,16,17,18,19,20,21,22], in addition to being environmentally friendly. These properties may vary according to the chemical composition and physical characteristics of the fibers. The main chemical constituent of an NLF is cellulose, which is responsible for the fiber’s stiffness. A higher amount of cellulose represents better mechanical properties. Another factor that influences the properties of the NLF is the part of the plant from which they are extracted, in addition to cultivation aspects, such as the age of the plant, climate and degradation processes [6,11,13,15].

Several studies have revealed an increase in the search for NLFs that can be used to reinforce polymeric composites, among which those found in the Amazon region stand out [23,24,25,26,27,28,29,30,31,32,33,34,35,36,37]; for instance, curaua [23,29], buriti [19,30], mallow [27,35], carnauba [38] and titica vine [39]. In addition to Amazon fibers, several other NLFs are being studied as possible reinforcements for polymer composites.

Pennas et al. [33] investigated tucum fibers for their basic characteristics, in terms of density, thermogravimetry, chemical composition and mechanical properties. These characterizations showed that tucum fibers present comparable results with other NLFs. The authors also point out that the similarity with sisal fibers may help future research regarding the potential use of tucum fibers as textile fibers.

Ding et al. [40] analyzed the chemical, thermal, mechanical and morphological properties of a new NLF, which was extracted from the stem of the rattan manau plant (*Calamus manan*) using mechanical separation. The results obtained revealed that this fiber can be used as reinforcement for thermoplastic composites with operating temperatures lower than 300 °C. Furthermore, the average tensile strength reached a value of 273.28 MPa and the scanning electron microscopy (SEM) analysis showed that the fiber presents a rough surface. According to the authors, these characteristics are beneficial for improving the mechanical properties and interfacial adhesion between fiber and matrix in composites. These results indicate the great potential of *Calamus manan* fibers as reinforcement in polymeric composites.

Kummar et al. [41] investigated the fundamental properties of a new plant fiber extracted from the bark of the *Acacia nilotica* tree. The fibers were extracted using the water maceration technique. According to the authors, it is necessary to perform a chemical treatment of the fiber surface to increase surface roughness. The chemical and physical analyses showed remarkable properties for its use as a reinforcement material in the production of composites for lightweight applications.

Vinod et al. [42] studied the properties of an NLF extracted from the stem of the *Cardiospermum halicababum* plant by the manual maceration process. The macerated fiber was analyzed for various physical, chemical, crystalline, thermal stability, tensile and morphological properties. The results obtained from these analyses indicated that *Cardiospermum halicababum* fiber has the advantage of replacing synthetic fibers in lightweight composite applications.

Another example of NLFs native to the Amazon region, which is found in large quantities and might have a potential for application as reinforcement in polymer composite materials, is extracted from the periquiteira plant known scientifically as *Cochlospermum orinocense*. The periquiteira plant, native to the dryland forest, is endemic to the Amazon region, occurring in the states of Mato Grosso, Maranhão and all the states of northern Brazil [43]. Traditionally, the fibers extracted from the plant’s stem are used to make ropes and reinforcements for basketwork, due to their high resistance. It is important to note that after the bark is removed for the extraction of the fibers, the periquiteira plant undergoes a sustainable regeneration process.

**Figure 1:**
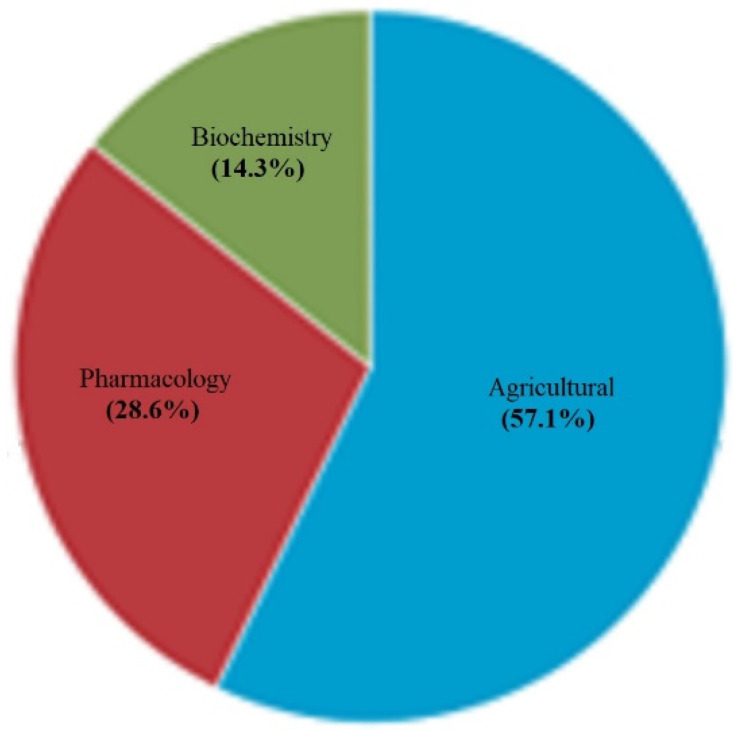
Interest areas of study about periquiteira plant according to Scopus database [44].

Research in the Scopus [44] database indicates that the periquiteira plant has been studied in the areas of agriculture, pharmacology, and biochemistry, as shown in Figure 1. However, to our knowledge, studies relating the use of fibers extracted from this plant to engineering applications have not yet been reported.

Therefore, for the first time, a broad investigation is made on the microstructural, morphological and mechanical characteristics of the periquiteira fiber, mainly regarding the characterization of crystallinity index (CI) and microfibril angle (MFA), which have not previously been reported in the literature. In addition, the ratio between tensile strength and the diameter of the periquiteira fibers is investigated, as well as the statistical validation of these data. Thus, this work is aimed to study the morphological, microstructural and mechanical characteristics of the periquiteira fibers, as well as to establish a correlation between the tensile strength of the fibers with their diametrical distribution pattern.

## 2. Materials and Methods

**Figure 2:**
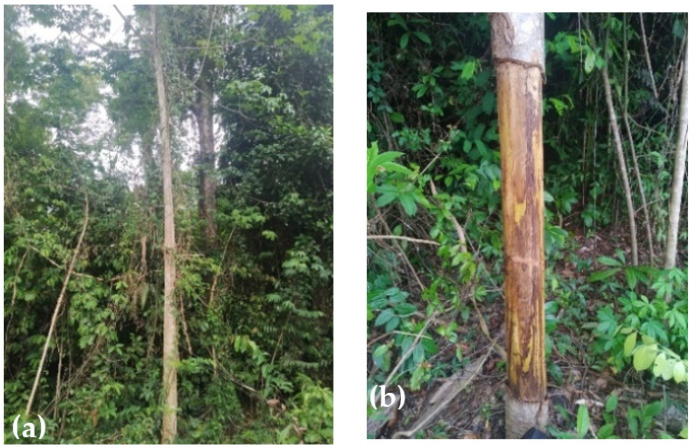

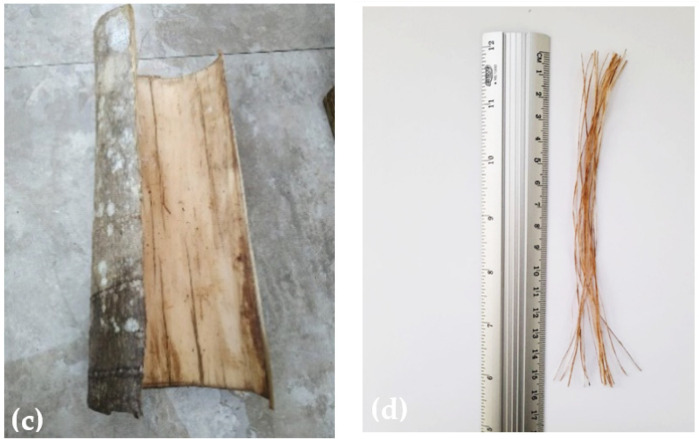
Periquiteira plant (**a**), bark extraction site (**b**), bark of the plant (**c**) and periquiteira fibers (**d**).

The periquiteira fiber was extracted from the stem of the plant, specifically found in the Amazon Cambium region, and supplied in the municipality of Baião, Pará, Brazil. Figure 2 illustrates the periquiteira plant, its bark extraction site, as well as the manually extracted fibers. The bark of the plant was immersed in water for 24 h to facilitate extraction of the fibers.

Table 1 shows the chemical composition of periquiteira fiber with referrer to cellulose and lignin contents.

### 2.1. Dimensional Characterization

#### Density

The apparent density, mass divided by volume, was obtained by the geometric method. To obtain the average diameter (d), each fiber was divided into five distinct points, uniformly separated, and then rotated by 90°. A new reading was carried out at the same five previous points. A total of three measurements were taken at each point, thus obtaining a statistical average. The equipment used was an Even optical microscope, model MDA 1300 (Future Win Joe, Xiasha, Hangzhou, China).

The length (*l*) of each fiber was measured using a 0.01 mm precision caliper, and its mass was weighed on a YMC electronic scale model, Chyo JK-200 (Kyoto, Japan), with a precision of 0.0001 g. To calculate the volume, it was considered that the fiber has a perfect cylindrical geometry using Equation (1) where *V_m_* is the average volume, *d* is the average fiber diameter and *l* is the average length:

(1) $$V_{m}=\frac{\pi d^{2}l}{4}$$

From the values obtained for mass (*m*) and the average volume of the fiber, it was possible to calculate the density (ρ) using Equation (2) and after that, the fibers were divided into diameter intervals and a correlation was made between the apparent density and the diametrical intervals.

(2) $$\rho =\frac{m}{V_{m}},$$

### 2.2. X-ray Diffraction (XRD)

The XRD analysis was performed on the Proto Manufacturing equipment, XRD Powder Diffraction System: the generator of 30 kV and 2 mA, Cu- Kα1 radiation, an angular step of 0.0149°, time interval of 0.5 s, sweep of 47 min and 2θ ranging from 5° to 60°.

The *CI* calculation followed the method described by Segal et al. [46]. It used the maximum intensity or the area of the peaks (1 0 1) and (0 0 2), which are associated with the amorphous and crystalline phases of the fibers. The value of *CI* was determined using:

(3) $$CI=\left(1-\frac{I_{1}}{I_{2}}\right)*100$$

In which *I*_1_ is the intensity of minimum (amorphous region) and *I*_2_ is the intensity of the diffraction maximum (crystalline region).

#### Microfibril Angle (MFA)

The MFA was evaluated by the method proposed by Cave [47] based on the cellulose (0 0 2) peak. Based on the XRD analysis, the MFA value is obtained from the relation between the Gauss curve and the first and second-order derivative curves. From this analysis, the parameter “T” is obtained, which is applied to the polynomial expression:


MFA = −12.19 × T^3^ + 113.67 × T^2^ − 348.40 × T + 358.09 (4)

### 2.3. Thermogravimetric Analysis (TGA)

To analyze the thermal stability of the fibers, TGA was performed in an equipment NETZSCH (Berlin, Germany) model STA 449 F3 Jupiter. The atmosphere used was nitrogen with a flow rate of 50 mL/min, a heating rate of 10 °C/min, and a temperature range from 20 to 500 °C.

### 2.4. Fourier Transform Infrared Spectroscopy (FTIR)

The FTIR analysis was performed in an equipment Bruker, model Vertex 70 v (Madison, WI, USA), using a mid-infrared range (4000–400 cm^−1^).

### 2.5. SEM Analysis

The periquiteira fiber surface morphology was analyzed by scanning electron microscopy (SEM) in a model Mira3 FEG 250 TESCAN microscope (Brno, Czech Republic) operating with secondary electrons at 5 kV and a work distance between 25 and 10 mm.

### 2.6. Tensile Tests

The determination of the mechanical properties obtained in 60 fibers was achieved using the tensile test. The preparation of each fiber specimen was performed according to ASTM C1557 [48]. Before the tensile test, the fibers were attached to a 90 m/g^2^ paper using epoxy adhesive. The tensile tests were performed in an EMIC DL 10,000 (Instron, São José dos Pinhais, Brazil) universal testing machine, with a 5 kN load cell. The tensile tests were carried out on specimens with 40-mm gage length using the displacement control at a rate of 0.5 mm/min. The cross-section of the fibers was considered circular, and their cross-section areas were used to calculate the tensile strength. Furthermore, the strain property was calculated from the load-displacement data, and apparent Young’s modulus was based on the slope of the stress–strain curve in the elastic region.

### 2.7. Statistical Analysis

The statistical validation of the data was performed using the analysis of variance (ANOVA), with a confidence interval of 95% (*p* < 0.05). The mean values were compared by the Tukey test.

## 3. Results

### 3.1. Dimensional Characterization

**Figure 3:**
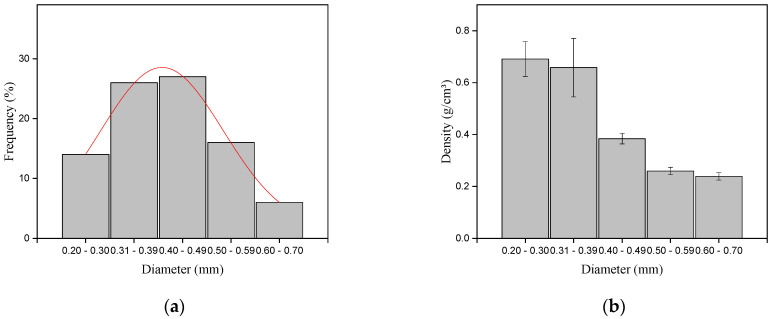
Distribution of the frequency of fibers (**a**) and the density relation (**b**) with periquiteira fiber diameter.

The distribution of fiber frequency in relation to the diameter interval is presented in Figure 3a and the variation of density with periquiteira fiber diameter is in Figure 3b.

It is possible to observe a higher amount of fiber in the diameter range of 0.4–0.49 mm, following an almost normal distribution pattern. This pattern suggests that the periquiteira fibers have a larger number of fibers with a medium diameter and only a smaller number of thick fibers. The value found for the fiber mean diameter was 0.50 mm. This result may be associated with the nature of the fibers, which being more compact, present a relatively smaller diameter. The mean density value, obtained from the geometric method, was 0.43 g/cm^3^. This value is lower when compared to the densities of other NLFs from the Amazon, such as guaruman and ubim [25,26,34]. It is also observed, in Figure 3b, that the mean density decreases as the diameter increases. This fact may be associated with a greater number of voids in the structure of larger-diameter fibers. The same pattern was observed for guaruman fiber, in which the thinner thickness fibers had fewer voids and open space pores and, consequently, higher density [26]. According to Moshi et al., [49] low-density fibers can be indicated for the production of lighter composites with high mechanical performance.

### 3.2. XRD Analysis

**Figure 4:**
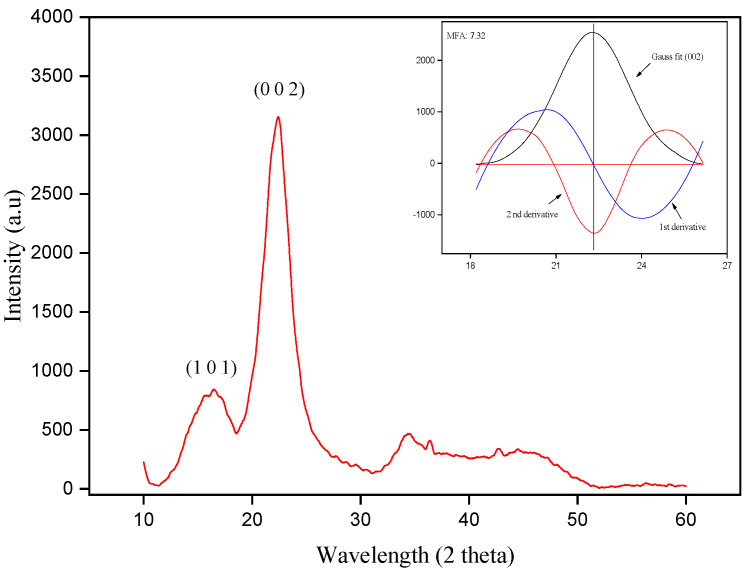
XRD pattern of the periquiteira fiber.

Figure 4 shows the diffractogram obtained from the periquiteira fiber. A halo is observed at an angle of 16.48° and a peak at 22.78°, referring to planes (1 0 1) and (0 0 2) which, respectively, indicate the presence of amorphous constituents and crystalline cellulose in the fiber. The crystallinity index value obtained from the periquiteira fiber was 70.49%. This result is comparable to other values reported from Amazonian NLFs, such as guaruman [25], buriti [30] and ubim [34].

The peak at 22.78°, which indicates the crystalline part of the fiber was used to evaluate the MFA and calculated as 7.32°. This value is similar to MFA values of other lignocellulosic fibers [25,34,38]. According to Junio et al., [38] low MFA values are considered to be favorable indicators of good mechanical properties, providing better fiber strength results.

### 3.3. TGA Analysis

**Figure 5:**
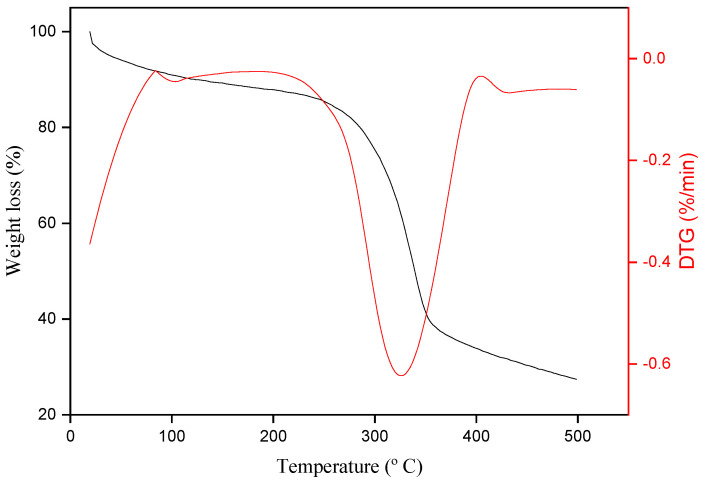
TG and DTG obtained for periquiteira fibers.

Thermogravimetric curve (TG) and its derivative (DTG) obtained for the periquiteira fibers are shown in Figure 5.

The TG curve shows that fiber mass reduction occurs within two temperature ranges, as noted by da Silva et al. [45]. Between RT and 120 °C, the first reduction occurs, related to the evaporation of water, which evidences the presence of the OH group in the structure of the fibers [50]. The second loss occurs between 250 and 320 °C, corresponding to the cleavage of glycosidic linkages of cellulose [50,51] beyond lignin degradation [52] that can occur by pyrolysis [50]. This same pattern was also observed by Moshi et al. [49] and Maache et al. [53]. The cellulose chain degradation is observed at temperatures close to 300 °C, due to the inert atmosphere [50] used in this experiment, a fact also observed by da Silva [45], Cunha et al. [54] and Moshi et al. [49]. After this temperature, no mass loss is observed, suggesting that beyond this stage, all components of the natural fiber were thermally degraded.

### 3.4. FTIR Analysis

**Figure 6:**
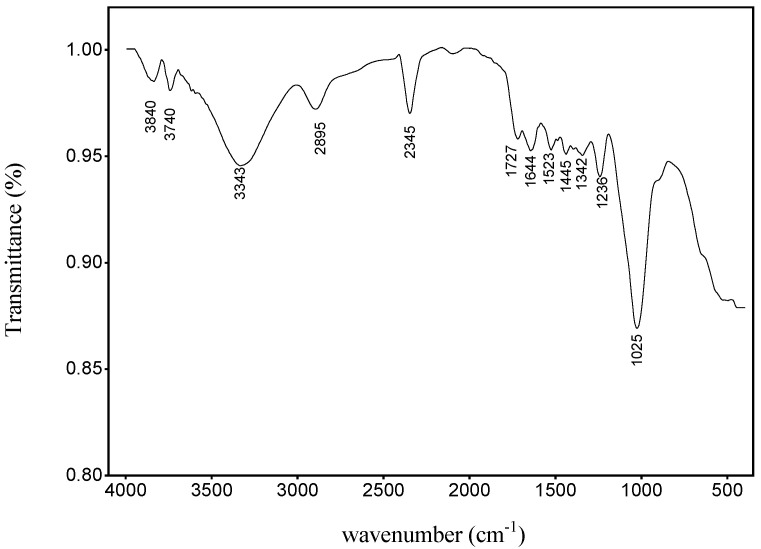
FTIR of the periquiteira fiber.

The FTIR spectrum of the periquiteira fiber is shown in Figure 6. This spectrum shows absorption bands of different chemical functional groups, which are present in the cellulose, hemicellulose and lignin structures. Between 3100 and 3800 cm^−1^ bands attributed to the stretching of the OH group are observed, evidencing the presence of cellulose and lignin [49,55]. The band at 2895 cm^−1^ corresponds to the elongation of the bond in CH, a constituent of cellulose [56]. At 2345 cm^−1^ the band referring to the alkyl chain denotes the presence of wax [56]. The band at 1727 is related to the stretching and bending of C=O in hemicellulose [57]. The absorbance at 1645 can be attributed to stretching C=C of the benzene ring referring to the lignin content [58]. At 1445 cm^−1^ it is an observed symmetric bending of CH_2_, which denotes the presence of cellulose [57]. The band at 1025 cm^−1^ denotes the presence of an aromatic ring related to the bending and vibration of C-H and CO [59].

### 3.5. SEM Analysis

**Figure 7:**
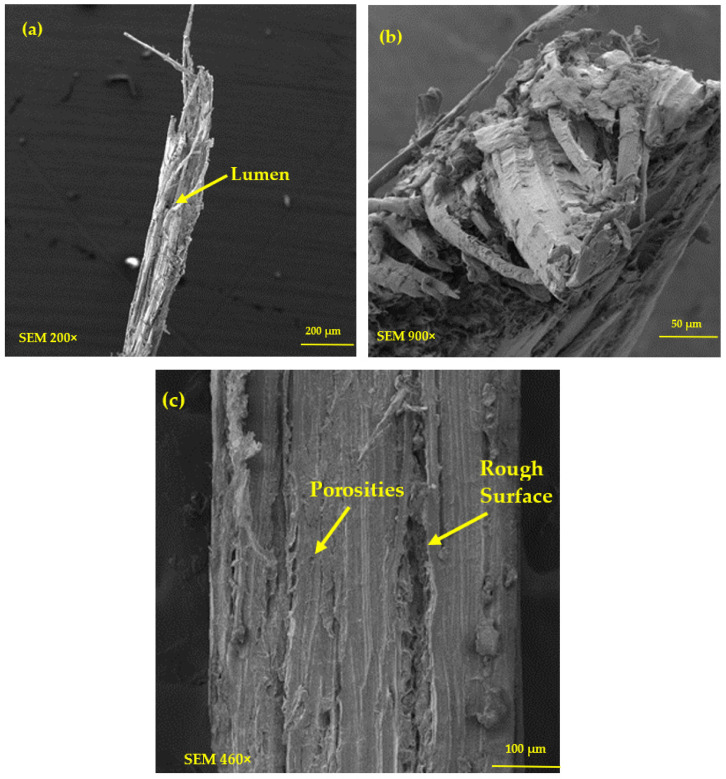
SEM surface morphology of the periquiteira fiber: (**a**) fiber cross-section with magnification of 200×; (**b**) fiber cross-section with magnification of 900× e (**c**) longitudinal section.

The morphology of the periquiteira fiber, analyzed by SEM, is shown in Figure 7. It is possible to observe in Figure 7a,b that the periquiteira fiber is composed of several thin bundles of fibrils. In addition, it presents flaws and irregularities along the fiber length. It is also still observed, in Figure 7c, that the fiber has a rough surface and the presence of porosity, beyond bundles of fibrils aligned in the direction of the length of the fiber. Manimaran et al. [55] also observed the presence of a rough surface, aligned bundles and pores. The presence of pores can affect the tensile strength property but, they may contribute to a better adhesion of the fiber in the matrix [53,59,60]. This factor may indicate good mechanical properties for periquiteira fiber-reinforced polymeric composites. Maache et al. [53] associated the improved mechanical anchoring of the *Juncus effusus* fiber in the matrix due to the observed surface roughness. The fiber morphological characteristics presented in Figure 7 may be compared with morphologies of different NLFs already reported in the literature [49,53,55,56,57,58,59,60].

### 3.6. Tensile Tests

**Figure 8:**
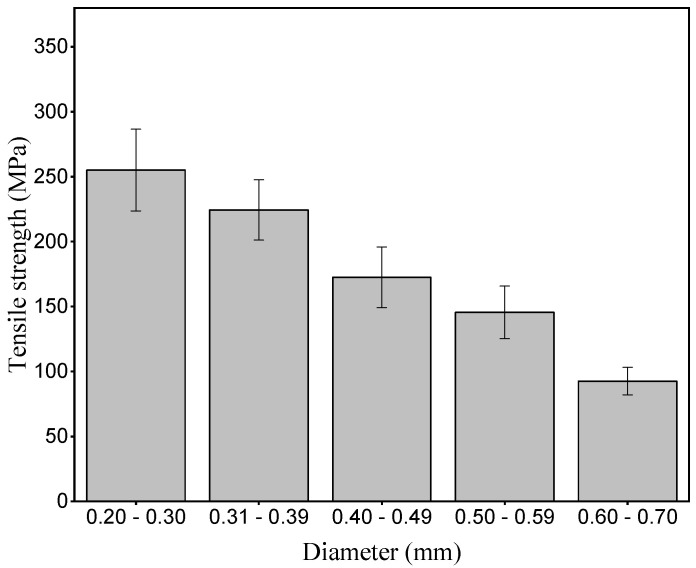
Tensile strength in relation to the diameter interval of the periquiteira fiber.

Figure 8 shows the graph of tensile strength in relation to the diameter interval of the periquiteira fiber.

The average value found for the strength of the periquiteira fiber was 178 MPa. This value is similar to that found by da Silva et al. [45]. However, it is higher when compared to the strength of some NLFs that have been investigated for possible applications in composite materials [49,53,55,56,57,58,59]. It is possible to observe a decrease in tensile strength with increasing diameter interval, suggesting that the tensile strength of the fibers is inversely proportional to the increase in the size of the fibrillar diameter. This correlation may contribute to the selection of more resistant fibers for manufacturing high-performance composites. Furthermore, it is suggested that thinner fibers may have higher cellulose contents to the detriment of thicker ones, which may also explain this inversely proportional correlation between tensile strength and fibrillar diametrical increase, however, this fact is still under study. The average value found for the smaller diameter interval was 255 MPa. This pattern may be related to the number of defects in the fiber structure, as observed in the micrographs, Figure 7, and also in the density pattern, Figure 3b, in relation to the diameter interval. Studies indicate that fibers with smaller diameters tend to present a uniform pattern with more compact microfibrils, fewer void spaces and, consequently, have greater strength [25,34]. However, in thicker fibers, there is a greater number of defects and microstructural porosity, which can cause a decrease in strength [61,62,63,64]. The resistance of the periquiteira fiber can be directly related to its high cellulose content and the low microfibrillar angle since high cellulose contents and greater angles confer greater mechanical resistance [34,55].

Marchi et al. [34], when characterizing the ubim fibers, found tensile strength values similar to those reported in the present study. Ribeiro et al. [35] found values of tensile strength for jute and mallow that were much higher than for the periquiteira fiber. However, the fiber studied might be considered lighter as an alternative for reinforcing polymeric matrix composites. In addition, the use of this new fiber can contribute to the development of more sustainable lightweight composites, since this fiber has a lower density when compared to other NLFs, such as jute and mallow [35], coir and banana [65].

**Figure 9:**
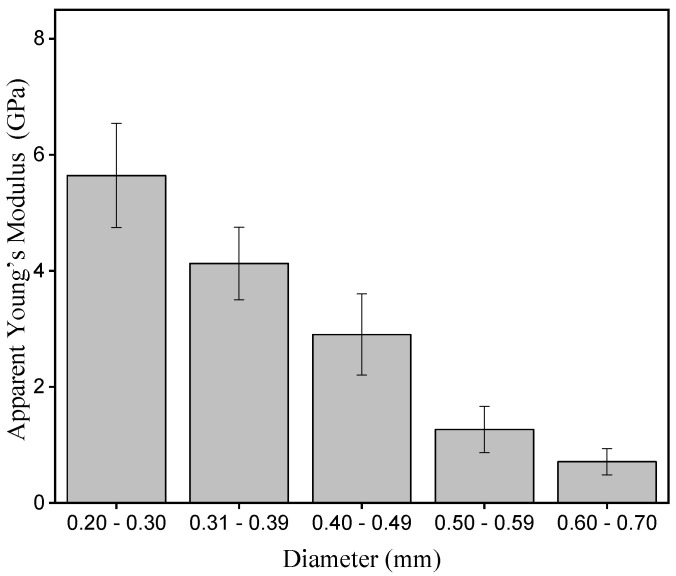
Apparent Young’s modulus in relation to the diameter interval of the periquiteira fiber.

Figure 9 shows the graph of the apparent Young’s modulus in relation to the periquiteira fibers diameter interval.

It is possible to observe that, with the increase of the diameter, there is a decrease in the average value of the apparent Young’s modulus. This fact indicates that the thinner periquiteira fibers present greater stiffness than the thicker fibers, suggesting that the fibers of smaller diameters have more compact fibril bundles. The inverse correlation of stiffness with diameter can be attributed to the fiber microstructure and also to the random rupture of microfibrils [66,67,68,69]. Similar behavior was observed by Zakikhani et al. [70] when studying the mechanical properties of bamboo fibers.

**Figure 10:**
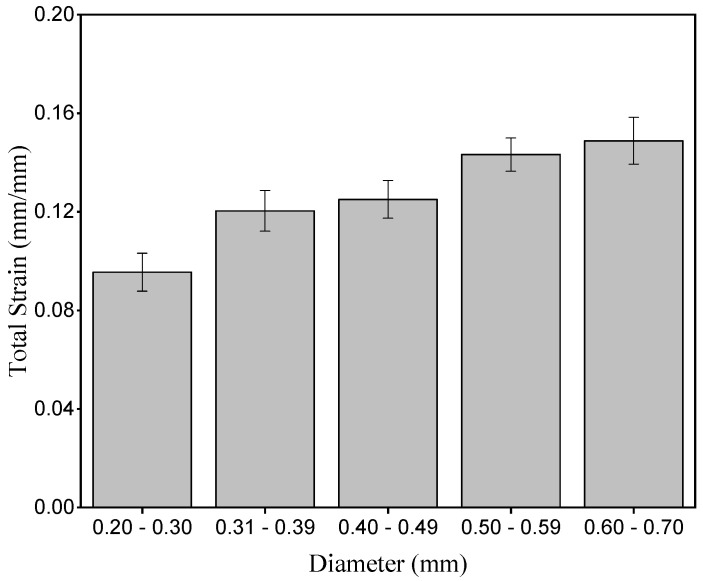
Strain in relation to the diameter interval.

Figure 10 presents the strain of the periquiteira fibers in relation to the diameter interval.

It is observed that the periquiteira fiber presents an increase in the average value of strain for fibers with greater diameter, confirming the results obtained for the apparent Young’s modulus shown in Figure 9. The greater amount of microfibrils and lower lignin contents [45] in the higher-diameter fibers may contribute to the increase in fiber strain.

### 3.7. Statistical Analysis

Statistical analysis of variance (ANOVA) of the mechanical characterization is presented in Table 2.

It is possible to observe that the calculated F values are higher than the critical F values for all properties. Thus, the hypothesis of equality between the average values of the properties for a significance level of 5% is rejected. Based on these results, it was necessary to perform Tukey’s test to evaluate whether increasing the diameter significantly changes the mechanical properties of the fibers. The results of the Tukey test are presented in Table 3.

The minimum significant difference (m.s.d) is a value that indicates which treatment differs in its mean values. When the absolute value of the difference between two means is equal to or greater than the m.s.d value, the means are considered statistically different. The m.s.d values obtained for the tensile strength, apparent Young’s modulus and total strain, were respectively 84.400, 2.276 and 0.030.

Thus, it was possible to observe that the diameter influences the mechanical properties of the fiber. The smaller diameter intervals have, in fact, higher tensile strengths and apparent Young’s modulus. The strain presents the smaller values in the thinner fibers. It is also believed that there is a directly proportional correlation between the density and the tensile strength of the periquiteira fibers, the fibers being denser, more resistant and with a smaller diameter. This can provide the production of composites with high mechanical performance and still light due to the average apparent density of this fiber.

## 4. Conclusions

The characterization of periquiteira fibers extracted from the stem of the plant, scientifically known as *Cochlospermum orinocense,* revealed that:

The fiber density measured by the geometric method was 0.43 g/cm^3^, which is among the lowest reported so far for natural lignocellulosic fibers (NLFs). In addition, it showed a tendency to decrease with increasing fiber diameter dimensions. The XRD analysis of the periquiteira fiber, permitted to calculate the crystallinity index (70.4%) and microfibrillar angle (7.32°), values that are associated with good mechanical properties of the fibers. The FTIR indicated the presence of different functional groups found in cellulose, hemicellulose, lignin and waxes, the main components of NLFs. The fiber morphology analysis revealed the presence of microfibrils, flaws and microstructural irregularities along its length, which are morphological characteristics similar to those observed in different NLFs. The mechanical characterization showed that the average values for strength and apparent Young’s modulus of the periquiteira fiber were 178 MPa and 2.92 GPa, respectively, and indicated a decrease in strength with increasing fiber diameter. Moreover, it was found that the resistance of the periquiteira fiber is directly associated with the fibrillar diameter, showing that the natural and intrinsic defects of the fiber directly affect its resistance, suggesting that thicker fibers have a greater number of defects and, therefore, are less resistant. The same pattern was observed in the density values, indicating that fibers with higher density, with fewer defects in their structure, presented better resistance. The average value of tensile strength, low fibrillar density and thermal results disclose that periquiteira fibers a promising NLF has the potential to be used to reinforce low-density polymeric composites, which can be applied in the civil construction and sports industry, replacing composites reinforced with synthetic fibers.

## Figures and Tables

**Table 1:** Periquiteira fiber chemical composition.

Periquiteira Fiber	Cellulose (%)	Lignin (%)	Reference
	60.15	15.03	[45]

**Table 2:** Analysis of variance for mechanical characterization in relation to the diameter interval.

Tensile Strength (MPa)						
Source	Sum of Squares	Degrees of Freedom	Mean of Squares	F (Calculated)	*p*-Value	F Critical
Between the groups	164,850.8	4	41,212.7	7.920	6.37 × 10−5	2.579
Inside the group	234,150.4	45	5203.342			
Total	399,001.2	49				
Apparent Young’s Modulus (GPa)						
Source	Sum of Squares	Degrees of Freedom	Mean of Squares	F (calculated)	*p*-value	F critical
Between the groups	164.845	4	41.211	10.888	2.93 × 10−6	2.579
Inside the group	170.327	45	3.785			
Total	335.173	49				
Total Strain (mm/mm)						
Source	Sum of Squares	Degrees of Freedom	Mean of Squares	F (calculated)	*p*-value	F critical
Between the groups	0.017	4	0.004	6.898	0.2 × 10−3	2.579
Inside the group	0.029	45	0.0006			
Total	0.047	49				

**Table 3:** Differences between the average values of the diameter intervals after applying the Tukey test.

Tensile Strength (m.s.d = 84.400)						Apparent Young’s Modulus (m.s.d = 2.276)					Total Strain (m.s.d = 0.030)				
	0.20–0.30	0.31–0.39	0.40–0.49	0.50–0.59	0.60–0.70	0.20–0.30	0.31–0.39	0.40–0.49	0.50–0.59	0.60–0.70	0.20–0.30	0.31–0.39	0.40–0.49	0.50–0.59	0.60–0.70
**0.20–0.30**	0	30.768	82.660	**109.628**	**162.569**	0	1.515	**2.739**	**4.376**	**4.932**	0	0.025	0.029	**0.048**	**0.053**
**0.31–0.39**	30.768	0	51.891	78.860	**131.800**	1.515	0	1.224	**2.861**	**3.416**	0.025	0	0.005	0.023	0.028
**0.40–0.49**	82.660	51.891	0	26.968	79.908	**2.739**	1.224	0	1.636	2.192	0.029	0.005	0	0.018	0.024
**0.50–0.59**	**109.628**	78.860	26.968	0	52.940	**4.376**	**2.861**	1.636	0	0.556	**0.048**	0.023	0.018	0	0.006
**0.60–0.70**	**162.569**	**131.800**	79.908	52.940	0	**4.932**	**3.416**	2.192	0.556	0	**0.053**	0.028	0.024	0.006	0

## Data Availability

Not applicable.
